# Screening and characterisation of proteins interacting with the mitogen-activated protein kinase Crmapk in the fungus *Clonostachys chloroleuca*

**DOI:** 10.1038/s41598-022-13899-3

**Published:** 2022-06-15

**Authors:** Binna Lv, Lele Fan, Shidong Li, Manhong Sun

**Affiliations:** Institute of Plant Protection, Chinese Academy of Agricultural Sciences, Beijing, 100193 China

**Keywords:** Biotechnology, Genetics, Microbiology, Molecular biology

## Abstract

*Clonostachys chloroleuca* 67-1 (formerly *C. rosea* 67-1) is a promising mycoparasite with great potential for controlling various plant fungal diseases. The mitogen-activated protein kinase (MAPK)-encoding gene *Crmapk* is of great importance to the mycoparasitism and biocontrol activities of *C. chloroleuca*. To investigate the molecular mechanisms underlying the role of Crmapk in mycoparasitism, a high-quality yeast two hybrid (Y2H) library of *C. chloroleuca* 67-1 was constructed, and proteins interacting with Crmapk were characterised. The library contained 1.6 × 10^7^ independent clones with a recombination rate of 96%, and most inserted fragments were > 1 kb. The pGBKT7-Crmapk bait vector with no self-activation or toxicity to yeast cells was used to screen interacting proteins from the Y2H library, resulting in 60 candidates, many linked to metabolism, cellular processes and signal transduction. Combined bioinformatics and transcriptome analyses of *C. chloroleuca* 67-1 and ΔCrmapk mutant mycoparasitising *Sclerotinia sclerotiorum* sclerotia, 41 differentially expressed genes were identified, which might be the targets of the *Fus3/Kss1*-MAPK pathway. The results provide a profile of potential protein interactions associated with MAPK enzymes in mycoparasites, and are of great significance for understanding the mechanisms of Crmapk regulating *C. chloroleuca* mycoparasitism.

## Introduction

*Clonostachys rosea*, a member of the order Hypocreales in the class Sordariomycetes, has a complex life style as a necrotrophic mycoparasite by killing other fungi and feeding on dead mycelia^1^. *C. rosea* is a promising biocontrol agent against numerous plant pathogenic fungi, including *Sclerotinia sclerotiorum*, *Rhizoctonia solani*, *Fusarium* spp. and *Botrytis cinerea*, and has achieved good results in greenhouse and field trials^1,2^. Multiple mechanisms, mycoparasitism, antagonism, competition, induced plant resistance, and secretion of cell wall-degrading enzymes are all involved in the biocontrol properties of the fungus^3–5^. In recent years, the functional genes of *C. rosea* have attracted much attention, and a number of genes, such as nonribosomal peptide synthetase gene *nps4*, transcription factor-encoding gene *crtf*, and cell wall biogenesis protein phosphatase gene *CrSsd1* were identified to be involved in fungal growth, conidiation, mycoparasitic ability, and biocontrol activity^6–8^. In the previous study, we sequenced and analysed the transcriptome of the highly efficient *C. chloroleuca* strain 67-1 (formerly *C. rosea* 67-1) mycoparasitising the sclerotia of *S. sclerotiorum*^9^, from which we identified the mitogen-activated protein kinase (MAPK)-encoding gene *Crmapk* that is orthologous to a *Fus3/Kss1* pathway member in *Saccharomyces cerevisiae*. *Crmapk* deficiency led to a reduced mycoparasitic ability to sclerotia and much lower biocontrol efficacy against soybean sclerotinia stem rot, indicating that *Crmapk* plays important roles in the biocontrol process of *C. chloroleuca*^10^. However, the molecular mechanism by which Crmapk regulates the mycoparasitism of *C. chloroleuca* remains unclear.

In eukaryotic organisms, transmitting signals into cells is a very complex process. When cells perceive external stimuli, a series of signal transduction pathways are initiated, and sequential cellular responses are triggered^11,12^. MAPK pathways comprising a MAPK kinase kinase (MAPKKK), a MAPK kinase (MAPKK), and a MAPK^13^ are important molecular systems that are directly connected with signal transduction and responses to diverse stresses, such as pheromones, osmotic pressure and growth factors^14–16^. In response to a stimulus, MAPKKKs phosphorylate and activate downstream MAPKKs, which in turn phosphorylate and activate MAPKs, and these enzymes phosphorylate specific downstream substrates, initiating cellular responses^17^. In *S. cerevisiae*, five MAPK pathways that regulate the biological processes of mating (Fus3/Kss1), filamentous growth (Kss1), cell wall integrity (Slt2), responses to high osmotic stress (Hog1) and ascospore formation (Smk1)^18,19^ have been identified. The functions of MAPK-encoding genes have been studied in different species, including some biocontrol fungi in the last decade. Gruber and Zeilinger^14^ found that deletion of MAPK-encoding gene *Tmk1* in *Trichoderma atroviride* altered the radial growth and conidiation of the fungus, inhibited the formation of infection structures, and reduced mycoparasitic ability to *R. solani* and *B. cinerea* hyphae. In *Coniothyrium minitans*, disruption of MAPK gene *CmSlt2* resulted in a lack of conidiation and a marked reduction of mycoparasitic ability to the sclerotia of *S. sclerotiorum*^20^. However, MAPKs may also act as negative modulators in response to stimuli and/or stresses. Mendoza-Mendoza et al*.*^21^ reported that deficiency of MAPK gene *Tvk1* induced the expression of mycoparasitism-related genes in *T. virens* in response to *R. solani*. In addition, the mutants exhibited enhanced biocontrol efficacy compared with the wild type strain and chemical fungicide.

During signal transmission, a series of proteins are activated responding to host and environmental stimulation. In plant pathogenic fungi, MAPKs interact with various kinds of proteins, such as transcription factor Ste12, MAPK phosphatase Msg5, and heat shock factor Sfl1^22–24^. However, there are no reports on proteins interacting with MAPK in biocontrol fungi, and the mechanism underlying MAPK-mediated regulation of mycoparasitism has not yet been clarified in mycoparasites.

Protein–protein interaction networks run through all biological and metabolic processes in living organisms. By using yeast two hybrid (Y2H) system, considerable interacting proteins and domains have been identified in plants, animals and fungi^19,25,26^. Y2H technique was firstly developed in *S. cerevisiae* by exploiting the transcription factor *GAL4* containing a separable DNA binding domain (BD) and a transcriptional activation domain (AD)^25^. Once prey proteins (library) fused to the DNA-AD domain interact with a bait protein (target) fused to the DNA-BD domain, *GAL4* promoters are activated and nutritional or antibiotic selectable markers are expressed as reporter genes, revealing putative interacting proteins^27^. Although some other methods such as coimmunoprecipitation and bimolecular fluorescence complementation are also employed^28^, high-throughput screening of Y2H libraries remains the most cost-effective and practical approach for protein–protein interaction studies in vivo.

To explore Crmapk-interacting proteins in *C. chloroleuca*, a high-quality Y2H library was generated using an in vivo recombination strategy, and 60 candidate interacting proteins involved in multiple biological processes were identified. To the best of our knowledge, this is the first report of proteins interacting with MAPK in *C. chloroleuca* and other hyperparasites identified by constructing a Y2H library. The findings provide valuable clues to the mechanisms by which Crmapk regulates mycoparasitism of *C. chloroleuca*.

## Results

### Quality of Y2H library of *C. chloroleuca* 67-1

**Figure 1:**
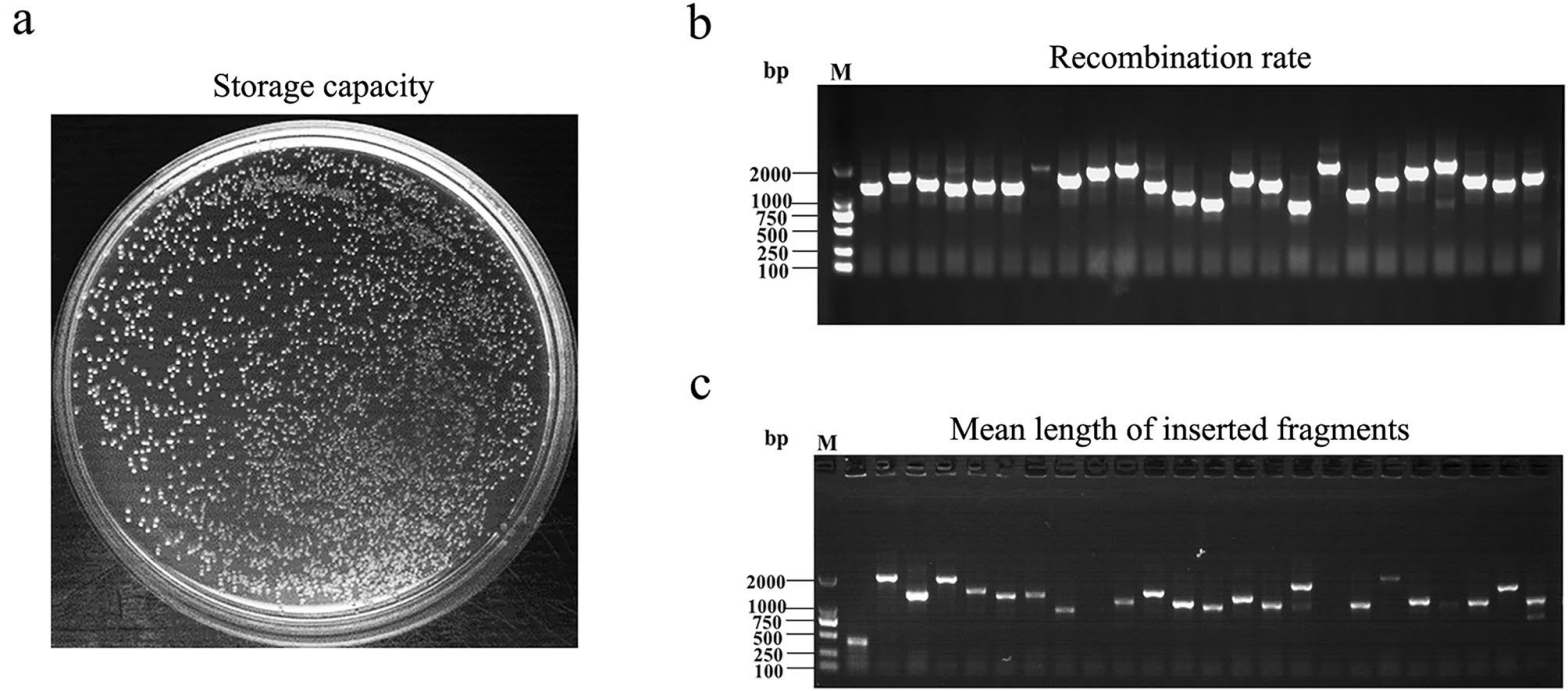
Analysis of the quality of the primary *C. chloroleuca* 67-1 library. (**a**) Determination of storage capacity. (**b**) Determination of recombination rate using primers pGADT7-F/R. (**c**) Determination of the mean length of inserted fragments from fungal colonies randomly selected by PCR amplification with primers pGADT7-F/R. *M* DNA 2000 marker, *bp* base pairs.

The mycelia of 67-1 strain under the induction of *S. sclerotiorum* sclerotia were collected for RNA extraction and construction of Y2H library. The quality of the Y2H library was evaluated, revealing 1.6 × 10^7^ primary clones (Fig. 1a), much higher than the generally required capacity of 1.0 × 10^6^ CFU/mL. The recombination rate of the library was 96% (Fig. 1b), and 24 randomly selected colonies were found to have an average insert size of ~ 1 kb (Fig. 1c), indicating that the Y2H library was of high quality and could be used for protein–protein interaction analysis.

### Construction of the pGBKT7-Crmapk bait vector and its auto-activation

**Figure 2:**
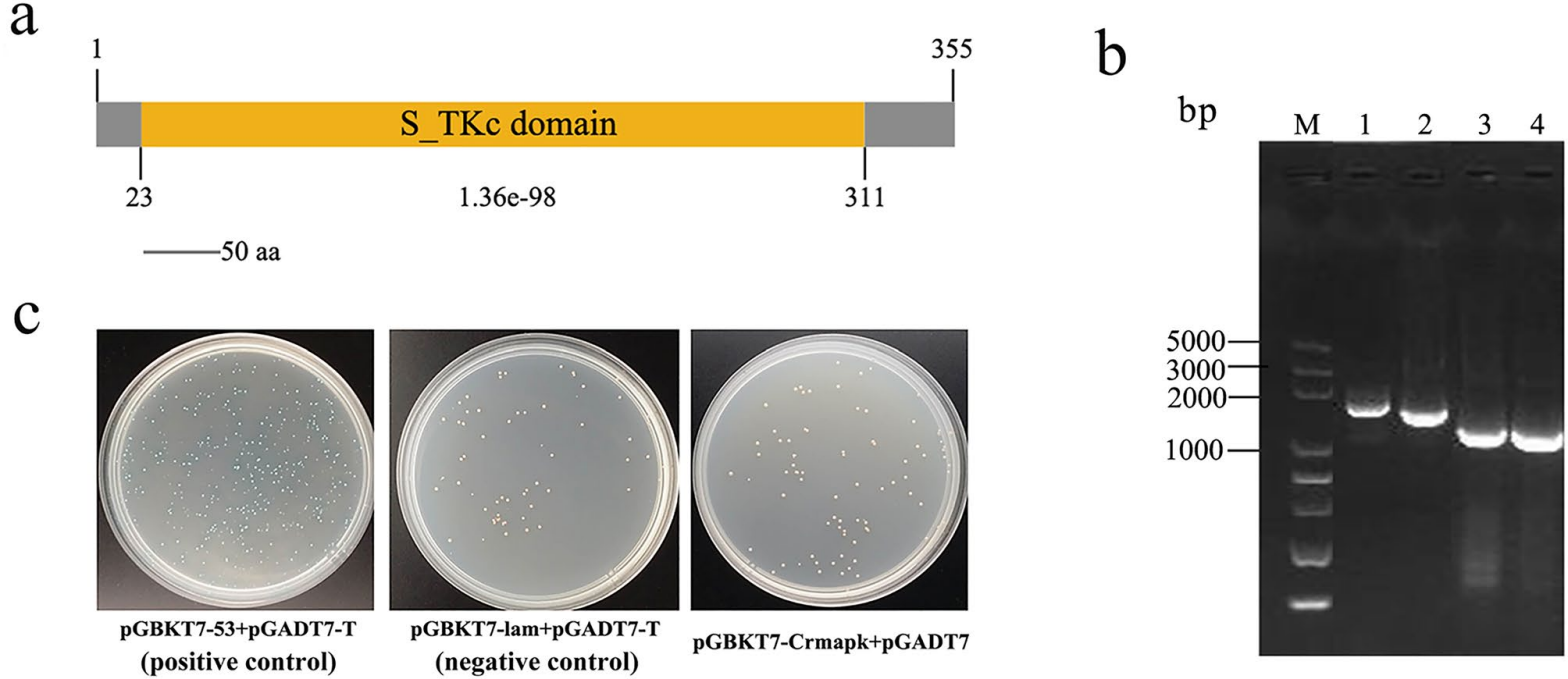
Construction of pGBKT7-Crmapk bait vector in *C. chloroleuca* 67-1. (**a**) Domain structure of Crmapk as annotated by SMART (http://smart.embl.de/). (**b**) Verification of the recombinant pGBKT7-Crmapk bait vector. M, DNA 5000 markers; Lanes 1–2, verification of the pGBKT7-Crmapk bait vector using vector primers T7/3′BD; Lanes 3–4, verification of the bait vector using target gene primers Crmapk-F/Crmapk-R. (**c**) Self-activation tests of the pGBKT7-Crmapk bait vector. The bait and empty vector pGADT7 were transformed into the Y2H Gold strain and cultured on SD/-Trp/X medium. pGBKT7-p53 and pGADT7-T were used together as a positive control, while pGBKT7-Lam and pGADT7-T served as a negative control.

The domain serine/threonine protein kinase (1068 bp) of the *Crmapk* gene was amplified from cDNA of *C. chloroleuca* 67-1 using specific primers Crmapk-F/Crmapk-R (Fig. 2a). The pGBKT7-Crmapk bait plasmid was successfully constructed and verified by PCR amplification using vector primers T7/3′BD and target gene primers Crmapk-F/Crmapk-R, and by DNA sequencing (Fig. 2b).

The availability of the pGBKT7-Crmapk bait vector was determined, and colonies containing the bait plasmid were white in colour on SD/-Trp/X (synthetic dropout medium lacking tryptophan and supplemented with 40 µg/mL X-α-gal) plates, while colonies containing the pGADT7-T and pGBKT7-53 positive control plasmids were blue, indicating that the pGBKT7-Crmapk bait could not autonomously activate the reporter genes (Fig. 2c). In addition, from visual analysis, fungal colonies containing bait and control plasmids had apparently the same sizes, suggesting that the constructed vector was not toxic to yeast cells. Thus, the pGBKT7-Crmapk bait vector could be used to screen the protein library for interacting proteins.

### Screening of Crmapk-interacting proteins

**Figure 3:**
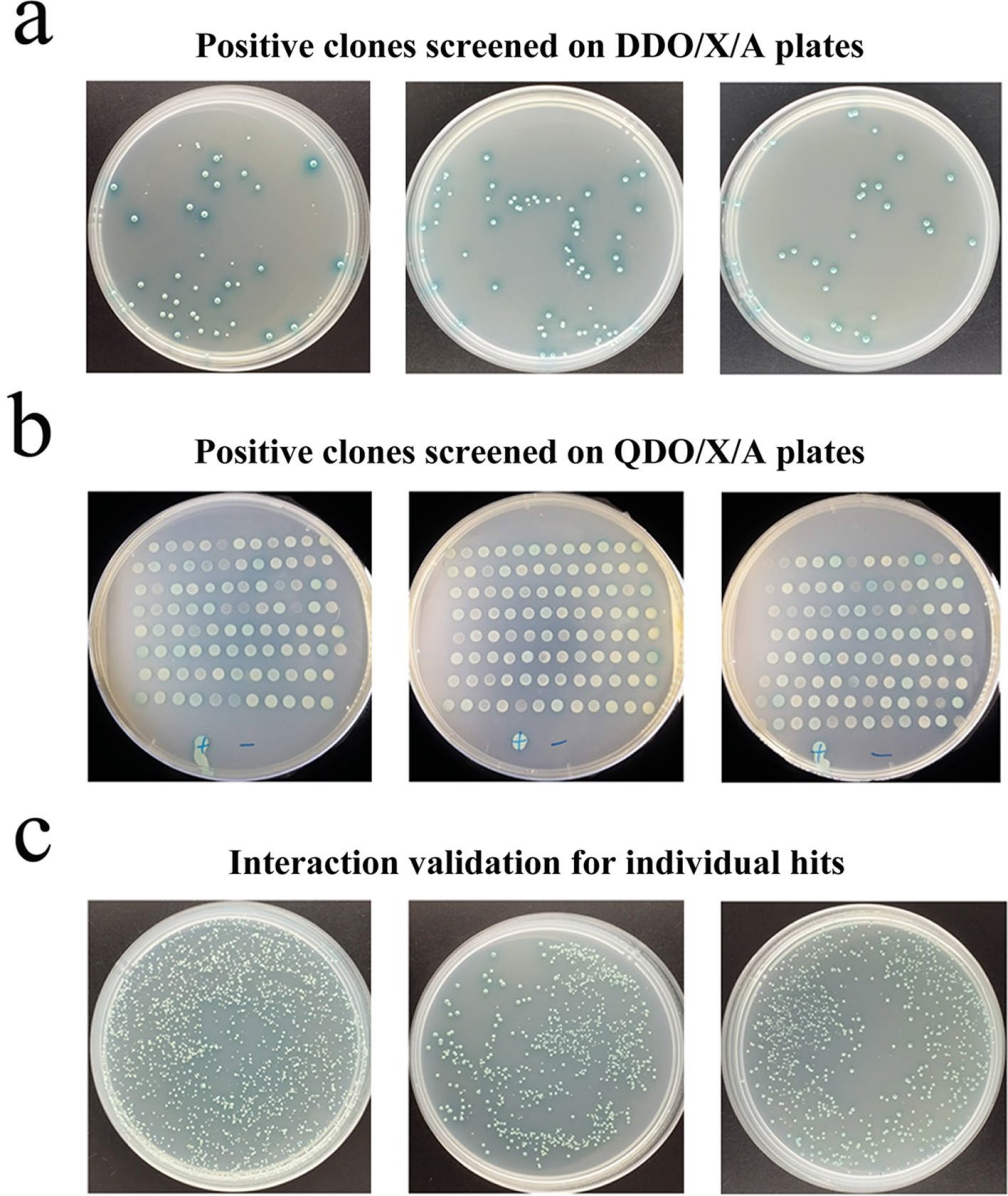
Screening and identification of putative interacting proteins in *C. chloroleuca* 67-1 via Y2H. (**a**) Positive clones screened on DDO/X/A plates. (**b**) Positive clones screened on QDO/X/A plates. “+” represents the positive control; “−” represents the negative control. (**c**) Interaction validation for individual hits. pGBKT7-53 and pGADT7-T plasmids were co-transformed into Y2H Gold cells as a positive control, while the pGBKT7-Lam and pGADT7-T served as a negative control. Three replicates were carried out for each treatment, and three plates were presented in the results.

After co-transformation of pGBKT7-Crmapk and prey plasmids, a total of 288 blue clones grew on SD/-Leu/-Trp/X-α-Gal/AbA (DDO/X/A) plates (Fig. 3a), among which 149 remained blue on high-stringency SD/-Ade/-His/-Leu/-Trp/X-α-Gal/AbA (QDO/X/A) plates (Fig. 3b), indicating that these clones might be genuine positives. All prey plasmids were verified by PCR amplification using vector primers pGADT7-F/R. These positive clones were separately co-transformed with pGBKT7-Crmapk, and finally, 80 blue clones emerged on QDO/X/A plates were obtained, which might express potential Crmapk-interacting proteins (Fig. 3c).

### Bioinformatic analysis of Crmapk-interacting proteins

The sequences of interacting proteins were aligned with the *C. chloroleuca* 67-1 genome database^29^, and 60 proteins interacting with Crmapk were identified (Supplementary Table S1). Among them, five genes presented higher interaction frequency than others, namely *NODE_505_4*, *NODE_320_3*, *NODE_606_27*, *NODE_522_14* and *NODE_689_14*, encoding a mitochondrial distribution and morphology protein, a putative C2H2 zinc finger domain-containing protein, a phospho-2-dehydro-3-deoxyheptonate aldolase, a CCR4-NOT transcriptional complex subunit, and a glucose-6-phosphate 1-dehydrogenase, among which the interaction frequencies of *NODE_505_4* and *NODE_320_3* were 8 and 7, respectively. Additionally, several interacting proteins were vital components of signal transduction pathways, such as COP9 signalosome complex subunit 5 (NODE_52_12), ubiquitin-conjugating enzyme E2 (NODE_405_44), transport proteins SEC23 and SEC31 (NODE_374_8 and NODE_558_28), and translocation protein SEC66 (NODE_29_18), indicating that these proteins might be involved in the similar pathways with Crmapk. SMART and Pfam database analyses showed that the domains of the 60 interacting proteins were diverse and mainly included translation protein SH3-like domains, protein kinase domains, C2H2 finger domains, peptidase S8/S53 domains, phosphofructokinase domains, and galactose mutarotase-like domains, implying that these domains might be involved in the interactions with Crmapk.

**Figure 4:**
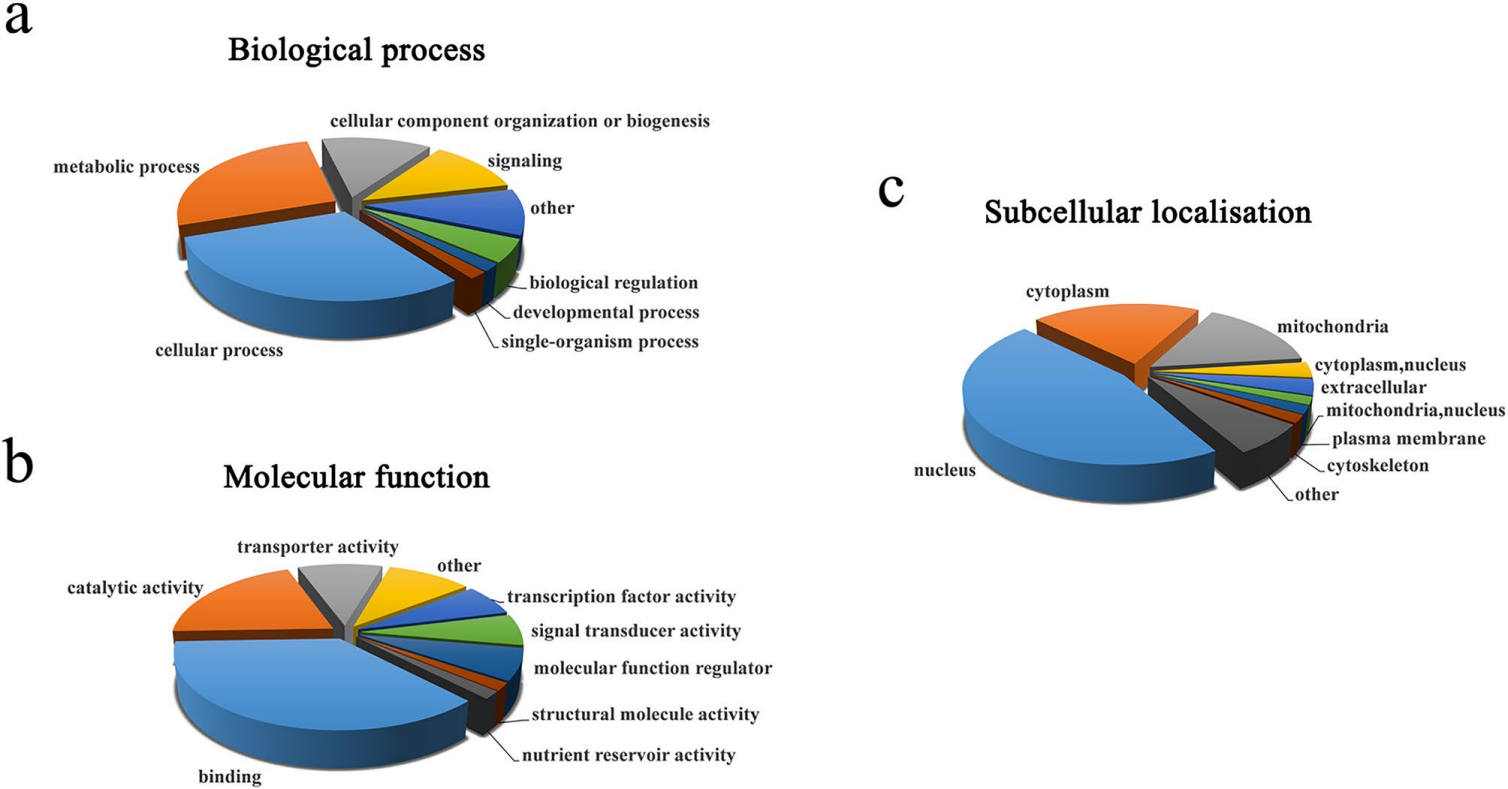
Gene Ontology functional classification of Crmapk-interacting proteins in *C. chloroleuca* 67-1. (**a**) Classification of interacting proteins based on biological process. (**b**) Classification of interacting proteins based on molecular function. (**c**) Subcellular localisation of interacting proteins. Blast2GO and WoLF SPORT Software were used for GO functional classification analysis.

To investigate the categories of MAPK-interacting proteins in *C. chloroleuca*, the number of identified proteins related to each Gene Ontology (GO) term was calculated according to GO annotation information. The results for biological process classification showed that interacting proteins involved in cellular processes were the biggest group, accounting for 30% of all candidate proteins. The second largest group contained proteins associated with metabolic processes, accounting for 27% of identified proteins (Fig. 4a). In molecular function category, 37% of the candidates had the characters of binding ability, while 30% of proteins with catalytic and transporter activities might also play important roles during mycoparasitic processes in this biocontrol fungus (Fig. 4b). Subcellular localisation of the interacting proteins was also analysed, and they were mainly distributed in the nucleus (46%), cytoplasm (20%) and mitochondria (15%) of fungal cells (Fig. 4c). These results indicate that the putative MAPK-interacting proteins are involved in diverse functions during *C. chloroleuca* mycoparasitism, especially cellular, metabolism processes, and binding, catalytic activities.

### Protein–protein interaction verified by GST pull-down assay

To further confirm the accuracy of the interactions identified by Y2H, six randomly selected putative interacting proteins NODE_320_3, NODE_439_51, NODE_505_4, NODE_1511_11, NODE_405_44 and NODE_606_27 that encoded a C2H2 zinc finger protein, an S8/S53 peptidase, a mitochondrial distribution and morphology protein, a NADH kinase pos5, a ubiquitin-conjugating enzyme E2, and a phospho-2-dehydro-3-deoxyheptonate aldolase, respectively, were assayed by the glutathione S-transferase (GST) pull-down method in vitro. The results showed that all the proteins could be eluted and were confirmed to interact with Crmapk (Fig. S1), indicating the Y2H results are reliable.

### Expression of *Crmapk*-interacting genes in *C. chloroleuca* 67-1 under the induction of sclerotia

To investigate mycoparasitism-related genes in *C. chloroleuca*, the transcriptome of the strain 67-1 associated with *S. sclerotiorum* sclerotia was sequenced and the differentially expressed genes 8 h, 24 h and 48 h after sclerotia induction were analysed^9^. Combining with these results, we found that 38 interacting genes were differentially expressed (|Log_2_ FC| ≥ 1 and *p* ≤ 0.05; Table 1), among which 15 genes (*NODE_15_2*, *NODE_171_30*, *NODE_176_9*, *NODE_198_20*, *NODE_2_38*, *NODE_201_16*, *NODE_374_8*, *NODE_38_86*, *NODE_486_20*, *NODE_507_12*, *NODE_52_12*, *NODE_525_9*, *NODE_558_28*, *NODE_606_27* and *NODE_98_38*) were upregulated by at least 1.2-fold at 8 h, similar to expression of *Crmapk* that especially highly expressed at 8 h during the mycoparasitic process of *C. chloroleuca* 67-1^10^. To explore the roles and mechanisms of *Crmapk* in *C. chloroleuca* mycoparasitism, the gene was deleted using gene homologous recombination strategy, and the transcriptome analysis of ΔCrmapk mutant mycoparasitising *S. sclerotiorum* sclerotia was performed before^10^. In this study, it showed that when *Crmapk* was deficient 11 putative interacting genes from the Y2H library were differentially expressed (|Log_2_ FC| ≥ 1 and *p* ≤ 0.05) based on the transcriptome profile of the *C. chloroleuca* mutant mycoparasitising sclerotia (Table 2), indicating that these genes might be involved in similar pathways to *Crmapk*. Among them, eight interacting genes were found to be differentially expressed in the wild type strain during parasitising sclerotia, including *NODE_320_3*, *NODE_439_51*, *NODE_29_18* and *NODE_17_25* encoding a C2H2 zinc finger protein, an S8/S53 peptidase, translocation protein SEC66, and *N*-acetyl-beta-d-glucosaminidase (NAGase), suggesting that they might account for the decreased mycoparasitic ability of *C. chloroleuca* 67-1. From above results, we speculate that all the differentially expressed genes maybe the targets of the *Fus3/Kss1*-MAPK pathway.

**Table 1:** Genes interacting with *Crmapk* that are differentially expressed in the transcriptome of *C. chloroleuca* 67-1 parasitising *S. sclerotiorum*.

Gene ID	Log_2_ FC (B8/A8)	Log_2_ FC (B24/A24)	Log_2_ FC (B48/A48)	Annotation
*NODE_115_3*	0.31	1.84	1.10	Acetyl-CoA carboxylase
*NODE_130_53*	0.55	0.25	1.04	6-Phosphofructokinase subunit alpha
*NODE_1331_7*	− 0.66	0.90	3.52	DNA polymerase eta subunit
*NODE_15_2*	2.33	1.60	2.67	Glucoamylase
*NODE_1511_11*	− 1.19	− 1.52	− 2.39	NADH kinase pos5
*NODE_1511_79*	− 0.38	0.41	1.08	Methionine aminopeptidase
*NODE_17_25*	− 0.14	− 0.38	1.87	*N*-acetyl-beta-d-glucosaminidase
*NODE_171_30*	1.38	1.42	1.52	Alpha-mannosidase
*NODE_176_9*	1.62	1.36	2.26	Protein transport protein sec-13
*NODE_19_9*	− 2.18	− 1.40	− 0.51	Hypothetical protein
*NODE_198_20*	1.79	0.80	− 0.39	SH3 domain-containing protein
*NODE_2_38*	1.22	0.53	0.31	Glutamate decarboxylase
*NODE_201_16*	1.78	2.15	1.93	Trihydroxynaphthalene reductase
*NODE_228_20*	− 0.76	− 0.57	1.22	Aldose 1-epimerase
*NODE_2303_57*	0.54	0.46	2.07	Flocculation protein FLO11
*NODE_254_7*	− 0.78	− 1.31	− 0.31	Serine/threonine-protein kinase srk1
*NODE_281_31*	− 1.59	− 0.49	0.09	Ribosome biogenesis protein NSA2
*NODE_29_18*	0.76	0.93	1.25	Translocation protein sec66
*NODE_320_3*	− 0.44	− 0.15	− 2.64	Putative C_2_H_2_ finger domain-containing protein
*NODE_374_8*	3.02	2.63	2.22	Transport protein SEC23
*NODE_38_86*	1.78	2.27	− 0.11	Aflatoxin B1 aldehyde reductase member 2
*NODE_403_52*	− 0.53	− 1.18	− 0.57	Zinc finger protein ADR1
*NODE_408_35*	− 0.52	− 0.23	1.10	Nucleoporin nsp1
*NODE_439_51*	− 1.10	1.52	− 1.52	Peptidase S8 and S53
*NODE_486_20*	3.33	3.51	3.30	Glucoamylase
*NODE_492_8*	− 1.45	− 1.56	− 0.78	Hypothetical protein
*NODE_505_4*	− 1.92	− 3.24	− 0.36	Mitochondrial distribution and morphology protein
*NODE_507_12*	1.91	1.55	1.96	Kinesin heavy chain
*NODE_514_22*	− 1.08	− 0.28	− 1.07	C_2_H_2_ type zinc finger domain-containing protein
*NODE_52_12*	2.50	2.06	1.34	COP9 signalosome complex subunit 5
*NODE_525_16*	0.73	− 0.26	1.12	DNA replication regulator SLD3
*NODE_525_9*	1.36	1.63	1.75	Asparagine synthase-like protein
*NODE_558_28*	1.48	0.26	2.53	Protein transport protein SEC31
*NODE_606_27*	2.19	1.61	1.18	Phospho-2-dehydro-3-deoxyheptonate aldolase
*NODE_69_11*	− 2.90	− 1.11	2.29	Glucosamine-6-phosphate deaminase
*NODE_98_38*	2.30	2.13	1.02	Elongation factor 2
*NODE_98_59*	− 1.10	1.53	1.87	Splicing factor 1
*NODE_98_63*	0.74	1.08	0.74	DENN domain-containing protein

Log_2_ FC, the abbreviation of Log [base 2] fold change, represents the relative expression of each gene (log2 value) between the control and treatments. The letter “A” represents the controls (samples without sclerotia) and “B” represents the treatments induced by *S. sclerotiorum* sclerotia. The samples of two groups were collected at 8 and 24 h, respectively, and the data of gene expressions were derived from our previous research^9^.

**Table 2:** Genes interacting with *Crmapk* that are differentially expressed in the transcriptome of the ΔCrmapk mutant parasitising *S. sclerotiorum*.

Gene ID	Log_2_ FC(B8/A8)	Log_2_ FC(B24/A24)	Annotation
*NODE_1511_11*	1.74	1.58	NADH kinase pos5
*NODE_17_25*	3.18	1.59	*N*-acetyl-beta-d-glucosaminidase
*NODE_19_9*	− 1.48	− 2.55	Hypothetical protein
*NODE_2303_85*	− 1.38	− 1.00	Golgi apyrase
*NODE_29_18*	− 1.18	− 0.06	Translocation protein sec66
*NODE_320_3*	− 4.70	− 4.29	Putative C_2_H_2_ finger domain-containing protein
*NODE_439_51*	− 0.94	− 2.57	Peptidase S8 and S53
*NODE_492_8*	− 2.93	− 3.00	Hypothetical protein
*NODE_505_4*	− 1.32	− 1.84	Mitochondrial distribution and morphology protein
*NODE_99_2*	0.64	1.03	DNA repair protein rad14
*NODE_990_5*	− 1.83	− 1.76	Hypothetical protein

Log_2_ FC, the abbreviation of Log [base 2] fold change, represents the relative expression of each gene (log2 value) between the control and treatments. The letter “A” represents the controls (samples without sclerotia) and “B” represents the treatments induced by *S. sclerotiorum* sclerotia. The samples of two groups were collected at 8 and 24 h, respectively, and the data of gene expressions were derived from our previous research^10^.

**Figure 5:**
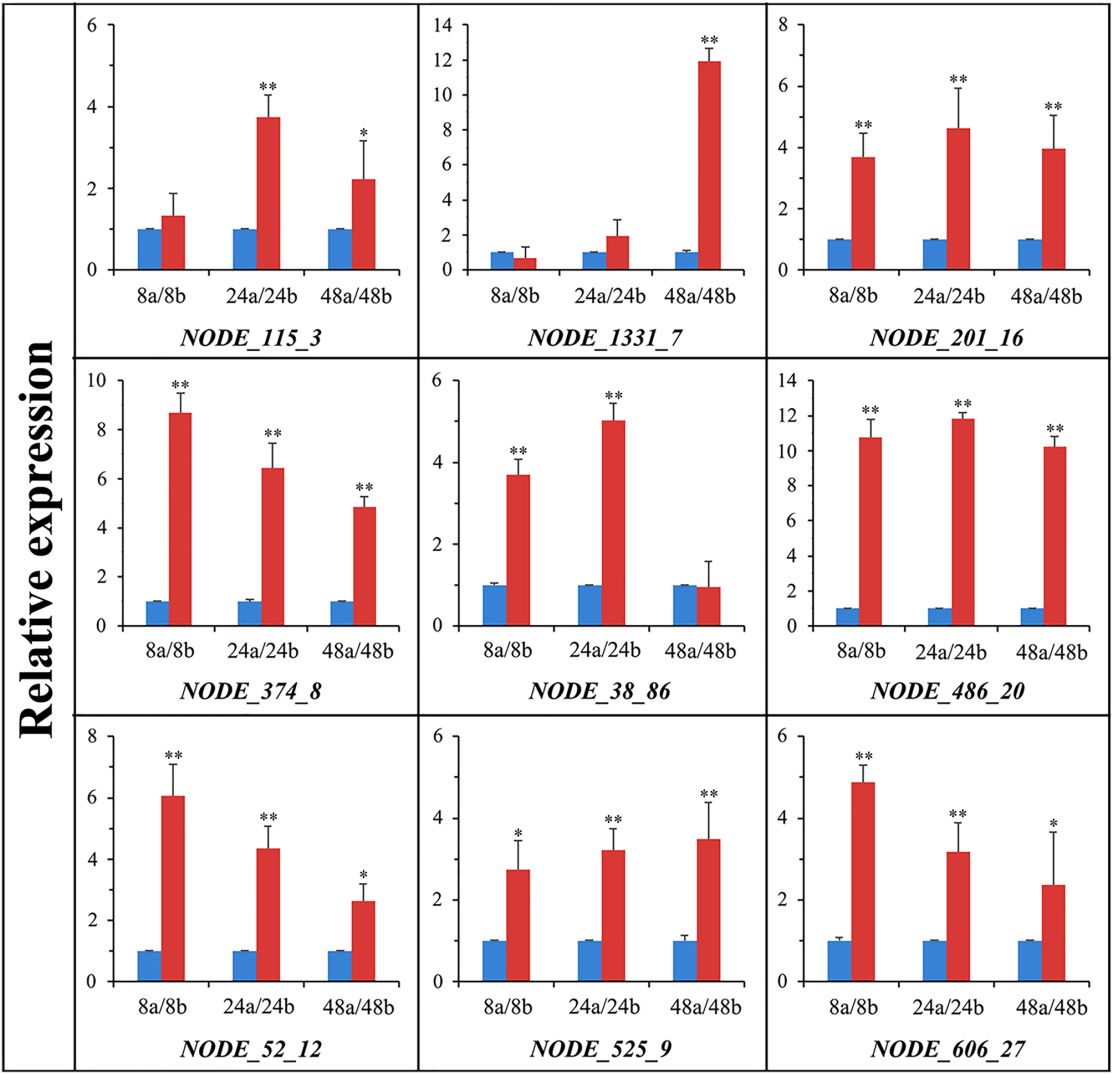
Expression levels of nine *Crmapk*-interacting genes in *C. chloroleuca* 67-1 under mycoparasitic conditions. The mycelia of 67-1 strain under the induction of sclerotia were collected at 8, 24 and 48 h. The letter “a” represents the controls (samples without sclerotia) and “b” represents the treatments induced by *S. sclerotiorum* sclerotia. Error bars indicate the standard deviation of three replicates. Statistical tests were carried out using Tukey’s test for multiple comparisons. Asterisks indicate statistically significant difference (*p* < 0.05).

In order to verify whether the transcriptome profiles were reliable for screening Crmapk-interacting proteins in *C. chloroleuca*, the relative expressions of nine differently expressed genes during mycoparasitising were analysed by using quantitative reverse transcription PCR (qRT-PCR), and the detailed information was shown in Supplementary Table S1. The results confirmed that these Crmapk-interacting genes encoding acetyl-CoA carboxylase, DNA polymerase eta subunit, trihydroxynaphthalene reductase, transport protein SEC23, aflatoxin B1 aldehyde reductase, glucoamylase, COP9 signalosome complex subunit 5, asparagine synthetase domain-containing protein, and phospho-2-dehydro-3-deoxyheptonate aldolase were consistent with the transcriptome data of *C. chloroleuca* 67-1 (Fig. 5). This suggests that the transcriptome profiles are reliable, and suitable for screening Crmapk-interacting proteins and probing the mechanisms by which Crmapk regulates mycoparasitism of *C. chloroleuca*.

## Discussion

MAPK signal transduction pathways are highly conserved in eukaryotic cells, and can modulate multiple cellular processes including embryogenesis, proliferation, differentiation, virulence and hyphal chemotropism through signal transduction from the surface of cells stimulated by extracellular factors^30^. In a previous study, it was confirmed that Crmapk played an important role in mycoparasitism of *C. chloroleuca*^10^. Crmapk is a protein kinase that acts as the central regulator of signal transduction pathways in *C. chloroleuca*, but its regulatory mechanisms are unknown. Proteins often work together with other protein partners to accomplish their essential functions in living organisms^31^. Therefore, characterisation of proteins interacting with Crmapk may provide information for understanding the molecular mechanisms by which Crmapk regulates mycoparasitism of *C. chloroleuca*. In previous studies, several MAPK-interacting proteins have been identified, including transcription factors Mst12 and Mcm1, and dual-specificity protein kinase Msg5 in pathogenic fungi^18,32,33^. In the present study, we constructed a high-quality Y2H library using the hyphae of *C. chloroleuca* induced by the sclerotia of *S. sclerotiorum*, from which proteins potentially interacting with Crmapk were screened and identified. To the best of our knowledge, this is the first report of identification of proteins interacting with MAPK in mycoparasites.

The Y2H system is widely used for the discovery of protein–protein interactions. This approach not only identifies putative interactions between two functional proteins, but also has great potential for high-throughput screening of uncharacterised proteins that bind to a given bait protein in large pooled cDNA libraries^26,34^. In the current study, we harvested mycelia from *C. chloroleuca* associating with *S. sclerotiorum* sclerotia rather than collecting samples from culture media, and thereby constructed a mycoparasitism-related cDNA library. Undoubtedly, a high-quality cDNA library is of great importance for obtaining reliable interaction data in Y2H analyses. In this study, the library capacity, recombination rate and size of inserted fragments that are important to the quality of cDNA libraries^35–37^ fulfilled the requirements and ensured the specificity and accuracy of the positive interactions. Although in some cases, Y2H analyses may generate a number of false-positives^34^, the proteins identified herein are highly likely to be related to the mechanism by which Crmapk regulates mycoparasitism of *C. chloroleuca*.

In many biological processes in eukaryotic organisms, MAPKs interact with other proteins to facilitate signal transmission. In the present study, 60 proteins putatively interacting with Crmapk were identified, most of which are new and perform diverse functions in filamentous fungi. Many of these proteins are related to signalosome complex, transcription factors and translocators, which involved in gene regulation, metabolism and signal transduction processes. Among them, C2H2-type zinc finger protein NODE_320_3 displayed high screening frequency. The C2H2 zinc finger family is a large group of transcription factors that play important roles in a variety of cellular functions, including gene expression, cell growth, proliferation, apoptosis and intracellular signal transduction^38^. Proteins may act as positive or negative regulators in MAPK signalling pathways to mediate cellular functions^39–41^. From the transcriptome of strain 67-1 under the induction of sclerotia, we found that *NODE_320_3* was differentially expressed, suggesting that it may contribute to the mycoparasitic ability of *C. chloroleuca*.

NODE_52_12 functions as a COP9 signalosome (CSN) complex. The CSN/COP9 signalosome has been shown to act in many pathways, mainly via ubiquitin degradation and signal transduction pathways^42,43^. Brockway et al.^44^ demonstrated that deficiency of the *csn* gene resulted in an inability to activate the MAPK pathway, which interrupted proliferation of *Caenorhabditis elegans*. In the present study, the *CSN* gene was also found to be markedly upregulated during the mycoparasitic process of *C. chloroleuca* 67-1^9^, indicating that the COP9 signalosome may play an important role in *C. chloroleuca* biocontrol activities.

Interacting proteins are more likely to be involved in similar biological processes and functions, and may be co-expressed in some circumstances^36^. In the transcriptome of *C. chloroleuca* 67-1 during the mycoparasitic process, *Crmapk* was especially highly expressed at 8 h, indicating its involvement in initiating *C. chloroleuca* mycoparasitism^10^. Combined with transcriptome analyses, the results showed that 15 interacting genes were upregulated during the early stages of the mycoparasitic process, consistent with the *Crmapk* expression profile. We speculate that these genes are likely to be closely connected with *Crmapk*, and involved in signal transduction following pathogen stimulation.

Peptidases of the S8/S53 family can degrade a broad range of substrates, including plant nematodes, and one such protein has been linked to the molecular mechanism of *C. rosea* infection of nematodes^45^. Pozo et al.^46^ found that overexpression of the serine protease encoding gene *tvsp1* in *T*. *virens* significantly increased its ability to protect cotton seedings against *R. solani*, and the gene was involved in the biocontrol process of the fungus. More interestingly, the S8/S53 protease gene family is in fact evolving under selection for increased gene copy number in *C. rosea*, which highlights its importance for mycoparasitism^47^. Herein, we found that peptidase S8/S53 gene *NODE_439_51* was differentially expressed in both wild-type and ΔCrmapk mutant strains during parasitism on *S. sclerotiorum*, implying that this peptidase might be involved in *C. chloroleuca* infection of pathogenic fungi.

Chitinolytic enzymes which are essential for catabolism of chitin primarily include chitinase, NAGase, and lytic polysaccharide monooxygenase^48^. Among them, NAGase that belongs to GH family 20 is capable of hydrolysing the terminal *N*-acetylglucosamine residues and exhibits a variety of biological activities^49^. The previous study of *T. harzianum* proved that the expression of the NAGase gene could be activated by three kinds of chitin-rich residues, chitosan, shrimp shell powder and mushroom wastes, sequentially enhanced the chitinolytic activity, which facilitated the biocontrol efficacy of *T. harzianum* against *F. oxysporum* in greenhouse^50^. In the present study, we found that NAGase gene *NODE_17_25* was differentially expressed both in the wild-type and ΔCrmapk mutant strains parasitising *S. sclerotiorum*, providing us a new insight into the biocontrol mechanism of *C. chloroleuca*.

Another interesting gene is *NODE_29_18* that encodes a translocation protein. Translocation proteins play auxiliary roles in recognition of precursors, and are closely related to signal transduction pathways^51^. In the present study, we found that this gene was differentially expressed in both isolates, indicating that the translocation protein may be closely related to MAPK pathways and involved in the mycoparasitic process of *C. chloroleuca*.

Based on the above findings, a regulatory network of *C. chloroleuca* mycoparasitism could be constructed from Crmapk and its interacting proteins. We speculate that once encountering a fungal host, Crmapk is highly expressed in *C. chloroleuca*, and simultaneously stimulates a series of related pathways to complete signal transduction and initiate the mycoparasitic process.

In conclusion, we identified putative MAPK-interacting proteins in *C. chloroleuca*, and the results may be applicable to other mycoparasites. The findings provide vital clues regarding molecular mechanisms by which Crmapk regulates mycoparasitism of *C. chloroleuca*, improve our knowledge of the mechanisms underlying biocontrol of *C. chloroleuca*, and help to the development of highly efficient biocontrol agents.

## Materials and methods

### Fungal strains and plasmids

*C. chloroleuca* 67-1 (ACCC 39160) was originally isolated from a vegetable yard in Hainan Province, China, using the sclerotia-baiting method^52^. *S. sclerotiorum* Ss-H (ACCC 39161) was separated from sclerotia-infected soybean stems in a field in Heilongjiang Province, China. Both strains were regularly cultured on potato dextrose agar (PDA) medium at 26 °C and maintained at 4 °C in the Biocontrol of Soilborne Diseases Lab of the Institute of Plant Protection, Chinese Academy of Agricultural Sciences.

### Construction of the Y2H library of *C. chloroleuca* 67-1

Strain 67-1 was incubated on PDA at 26 °C for 10 days, spores were washed with sterile water and adjusted to 1 × 10^7^ spores/mL, and spore suspensions were smeared evenly on a PDA plate and covered with cellophane. Uniformly sized sclerotia were placed onto the surface of strain 67-1 plates evenly after culturing for 48 h, and the mycelia of *C. chloroleuca* 67-1 during mycoparasitic process and vegetative growth were collected, respectively, at 24 h and placed immediately in liquid nitrogen. Each treatment included five replicates.

Total RNA was harvested using TRIzol reagent (Invitrogen, California, USA), then treated with DNase I (TransGen, Beijing, China) to eliminate contaminated genomic DNA. The purity and integrity of total RNA were determined using a NanoDrop 1000 instrument (Thermo, Waltham, USA) and agarose gel electrophoresis, respectively. The isolation and purification of poly(A) mRNA from total RNA were carried out using an Oligotex mRNA Midi Kit (QIAGEN, Hilden, Germany) according to the manufacturer’s instructions. The cDNA library was acquired using a CloneMiner II cDNA Library Construction Kit (Invitrogen) according to the manufacturer’s protocols.

Following normalisation and short fragment removal, the purified cDNA and linearised pGADT7-DEST vector (prey plasmid), including three different reading frames to confirm the correct expression of all proteins, were co-transformed into the Y187 yeast strain using Yeastmaker Yeast Transformation System 2 (Clonetech, Shiga, Japan). After culturing on synthetic defined medium lacking leucine (SD/-Leu) to select transformants, a series of dilutions of the transformed mixture were also spread on SD/Leu plates to calculate the transformation efficiency and isolate separate colonies. After culturing at 30 °C for 3–6 days, positive transformants were harvested to form an Y2H library. A hemacytometer (Thermo, Waltham, USA) was used to measure the cell density of the Y2H library to ensure that the library capacity was no less than 1.0 × 10^6^ CFU, which is essential for a high-quality Y2H library. To check insert sizes and the recombination rate of the Y2H library, 24 colonies were randomly picked out and amplified by PCR using primers pGADT7-F/R (Table 3). The primary library was retransformed into competent *Escherichia coli* DH10B, and the plasmids were harvested and stored at − 80 °C.

**Table 3:** Primers used in this study.

No	Primer name	Sequence (5′–3′)
1	Crmapk-F	CATGGAGGCCGAATTCATGTCTCGATCAACTCAGCCCAGC
2	Crmapk-R	GCAGGTCGACGGATCCTCATCGCATGACCTCCTGGTAGAT
3	T7	TAATACGACTCACTATAGGG
4	3′BD	TTTTCGTTTTAAAACCTAAGAGTC
5	pGADT7-F	TAATACGACTCACTATAGGGCGAGCGCCGCCATG
6	pGADT7-R	GTGAACTTGCGGGGTTTTTCAGTATCTACGATT
7	NODE_115_3-F	AGAAGCACCCGTCACAATTG
8	NODE_115_3-R	GACACCGCTCACCATTTCAG
9	NODE_1331_7-F	ACTTTTGGCAAGTCAACCTCA
10	NODE_1331_7-R	GGTTACCAAGCCCCTCTAGG
11	NODE_201_16-F	AAGGAGAACAAGCGTCTGGA
12	NODE_201_16-R	GGGGAGGTGATCGAGACAG
13	NODE_374_8-F	GCAAAGCAGGTCCAAGATATGT
14	NODE_374_8-R	CTCGAGAAGTCCCACAGCAA
15	NODE_38_86-F	GGGACAATACTGCAGACAAGG
16	NODE_38_86-R	TGTACATGCCTTGGTAGACTGT
17	NODE_486_20-F	ATGAACATGACATGCCGAGC
18	NODE_486_20-R	GGTCTTGTCTGTGTCCTCGA
19	NODE_52_12-F	GTATTTGGACCAGTGCCGTG
20	NODE_52_12-R	CTCTTGTAGTCGGCAGGGTA
21	NODE_525_9-F	CACACAGACGGCCCCATC
22	NODE_525_9-R	GCTCGTTGTCAGCCAGTAAC
23	NODE_606_27-F	AACCGCATCAAACTTCCTGC
24	NODE_606_27-R	CCATAGCTTCTCATCGCCGG
25	EF1-F	TCGATGTCGCTCCTGACT
26	EF1-R	AGCGTGACCGTTTATTTGA

### Construction of the pGBKT7-Crmapk bait vector

To screen interacting proteins from the *C. chloroleuca* Y2H library, the pGBKT7-Crmapk bait vector was constructed. Using *C. chloroleuca* cDNA as a template, the full-length coding sequence (CDS) of *Crmapk* (GenBank accession number: KY701731) was amplified with specific primers Crmapk-F and Crmapk-R (Table 3), containing restriction sites *Bam*HI and *Eco*RI. The resulting PCR product was purified and inserted into bait vector pGBKT7 harbouring the *GAL4* DNA-binding domain (BD). The recombinant pGBKT7-Crmapk bait plasmid was verified by double restriction enzyme digestion and DNA sequencing (TSINGKE, Beijing, China).

### Auto-activation tests of the pGBKT7-Crmapk bait vector

Bait vector pGBKT7-Crmapk and empty vector pGADT7 were separately transformed into the Y2H Gold strain using the PEG/LiAc-mediated method. The vectors pGBKT7-p53 and pGADT7-T co-transformed were used as positive control, while pGBKT7-Lam and pGADT7-T were used as negative control. Transformants were grown on SD/-Trp (SD medium lacking tryptophan), SD/-Trp/X and SD/-Trp/X/A (SD-Trp supplemented with 40 µg/mL X-α-gal and 200 ng/mL aureobasidin A) plates at 30 °C for 3–5 days. Following the growth of white colonies on SD/-Trp and SD/-Trp/X plates, and no colony growth on SD/-Trp/X/A plates, the bait was verified without auto-activation. In addition, if the bait was toxic, the colonies containing the bait plasmid were obviously smaller than the control treatments. Only the bait vectors that showed no auto-activation activity or toxicity were used for Y2H screening.

### Screening of Crmapk-interacting proteins

In order to screen proteins interacted with Crmapk, bait vector pGBKT7-Crmapk and the Y2H AD library were co-transformed into the Y2H Gold strain using the yeast mating method. The mating culture was plated on DDO/X/A agar plates at 30 °C for 3–5 days, and all blue colonies on DDO/X/A agar plates were patched onto higher stringency QDO/X/A agar plates. To further verify the interactions, the prey plasmids of each primary interacting protein were rescued from yeast strains and separately co-transformed with pGBKT7-Crmapk into the Y2H Gold yeast cells. The transformants were then retested on DDO/X/A and QDO/X/A media at 30 °C for 3–5 days, after which resulting blue colonies were considered to be potential positive clones. At the same time, the pGBKT7-53 and pGADT7-T plasmids were co-transformed into Y2H Gold cells as a positive control, while the pGBKT7-Lam and pGADT7-T served as a negative control. Three replicates were carried out, and all positive clones were identified by DNA sequencing using primers pGADT7-F/R for the T7 promoter.

### GST pull-down assay

The DNA fragment of Crmapk was cloned into the vector pGEX-4T-1 (GE Healthcare, Chicago, United States) to generate the Crmapk-GST fusion protein. Six interacting proteins NODE_320_3, NODE_439_51, NODE_505_4, NODE_1511_11, NODE_405_44 and NODE_606_27 localising in the nucleus, cytoplasm and mitochondria basing on bioinformatics analyses were selected randomly and corresponding his-labelled proteins were constructed in the pCZN1 (Zoonbio, Nanjing, China) vector separately. GST, Crmapk-GST, NODE_320_3-his, NODE_439_51-his, NODE_505_4-his, NODE_1511_11-his, NODE_405_44-his and NODE_606_27-his plasmids were expressed in the *E. coli* BL21 cells (Sangon, Shanghai, China). The cells were lysed in lysis buffer (50 mM Tris, pH 8.0, 50 mM NaCl, 1 mM PMSF) with a sonicator (Scientz, Ningbo, China) and centrifuged at 13,000*g*, for 10 min. The supernatants were transferred to a 1.5 mL tube and stored at − 70 °C. The GST and Crmapk-GST supernatants were mixed with 30 μL glutathione Sepharose beads (GE Healthcare) and incubated at 4 °C for 2 h, and then the recombinants of Crmapk-GST and GST bound to the Sepharose beads were incubated with the cell lysate of *E. coli* that contained NODE_320_3-his, NODE_439_51-his, NODE_505_4-his, NODE_1511_11-his, NODE_405_44-his, and NODE_606_27-his at 4 °C. After treated for 4 h, the beads were washed with buffer (50 mM Tris, pH 8.0, 50 mM NaCl, 1 mM PMSF, 1% Triton X-100) five times and the eluted proteins were analysed by immunoblot with monoclonal anti-His and monoclonal anti-GST antibodies.

### Bioinformatics analyses of Crmapk-interacting proteins

To identify the corresponding interacting proteins, all sequences obtained from the Y2H assays were analysed using BLAST (https://blast.ncbi.nlm.nih.gov). Homologous proteins were also identified in *S. cerevisiae* by BLAST. Next, GO analysis (http://amigo.geneontology.org/amigo) was performed to probe gene functional classification basing on biological process, molecular function and subcellular localisation. KEGG pathway analysis (https://www.kegg.jp/) was conducted to investigate high-level functions and pathways from molecular-level information. Protein function and interaction networks of the identified interacting proteins were generated using the UniProt database (http://www.uniprot.org/) and STRING (https://string-db.org/). InterPro (http://www.ebi.ac.uk/interpro/) and SMART (http://smart.embl.de/) databases were used to predict protein domains encoded by the prey fragments.

### qRT-PCR detection of Crmapk-interacting genes

Nine differentially expressed interacting genes (*NODE_115_3*, *NODE_1331_7*, *NODE_201_16*, *NODE_374_8*, *NODE_38_86*, *NODE_486_20*, *NODE_52_12*, *NODE_525_9*, *NODE_606_27*) were selected randomly and their expression levels during mycoparasitism on *S. sclerotiorum* sclerotia were determined by qRT-PCR as previously described^8^. The letter ‘a’ represents the controls (samples without sclerotia) and ‘b’ represents the treatments by *S. sclerotiorum* sclerotia. The mycelia of *C. chloroleuca* 67-1 under the induction of sclerotia were collected at 8, 24 and 48 h, and total RNA was extracted using TRIzol reagent following the manufacturer’s instructions. The cDNA was prepared from total RNA using a cDNA FastQuant RT Kit (TIANGEN, Beijing, China), and the expression of Crmapk-interacting genes derived from the Y2H library was investigated using a Bio-Rad IQ 5 Real-Time System (Bio-Rad, California, USA) and SYBR Premix Ex Taq (Takara, Dalian, China). Elongation factor gene *EF1* (GenBank accession number: KP274074) was used as an internal reference to normalise gene expression, and the primers used for qRT-PCR determination of the candidates were listed in Table 3. Amplification by qRT-PCR involved heating at 95 °C for 30 s, followed by 40 cycles at 95 °C for 5 s and 55 °C for 30 s. After PCR amplification, fluorescence values were measured every 0.5 °C from 55 to 95 °C, and the relative expression levels of Crmapk-interacting genes were calculated using the 2^−∆∆Ct^ method^53^. All reactions were performed in triplicate. Statistical software SPSS 2.0 (Chicago, IL, USA) was used for ANOVA. Statistical tests were carried out using Tukey’s test for multiple comparisons and a *p* < 0.05 was considered statistically significant.

## Supplementary Information

Supplementary Figures.

Supplementary Table S1.
