# Supplementary material for: Screening and characterisation of proteins interacting with the mitogen-activated protein kinase Crmapk in the fungus Clonostachys chloroleuca
Source: Sci Rep. 2022 Jun 15;12:9997. doi: 10.1038/s41598-022-13899-3 (PMC9200739; doi:10.1038/s41598-022-13899-3)

**Figure** **S1.** Protein-protein interaction verified by GST pull-down assay. **(a)** Interaction between Crmapk and NODE_320_3-his. **(b)** Interaction between Crmapk and NODE_439_51-his. **(c)** Interaction between Crmapk and NODE_505_4-his. **(d)** Interaction between Crmapk and NODE_1511_11-his. **(e)** Interaction between Crmapk and NODE_405_44-his. **(f)** Interaction between Crmapk and NODE_606_27-his.


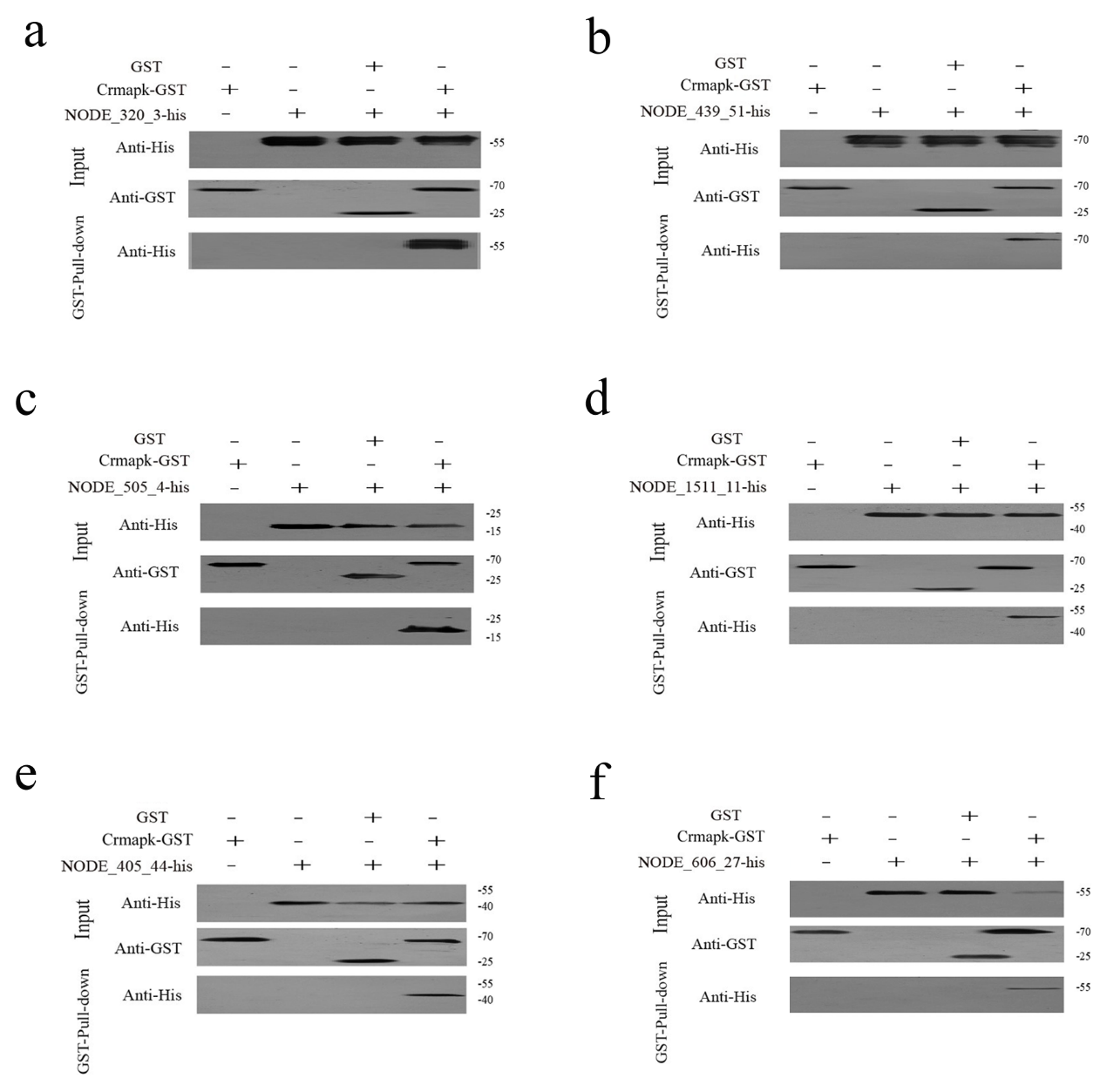

Supplement: Supplementary file 1 — Supplementary Figures. [file 41598_2022_13899_MOESM1_ESM.docx]
