# Supplementary material for: Screening and characterisation of proteins interacting with the mitogen-activated protein kinase Crmapk in the fungus Clonostachys chloroleuca
Source: Sci Rep. 2022 Jun 15;12:9997. doi: 10.1038/s41598-022-13899-3 (PMC9200739; doi:10.1038/s41598-022-13899-3)
Supplement: Supplementary file 2 — Supplementary Table S1. [file 41598_2022_13899_MOESM2_ESM.docx]

**Table S1.** The information of the proteins interacting with Crmapk by Y2H assay.

| **No.** | **Gene ID** | **Gene length** | **Protein name** | **Domain description** | **Frenquency** |
| --- | --- | --- | --- | --- | --- |
| 1 | *NODE_1038_55* | 1642 | Predicted protein | / | 1 |
| 2 | *NODE_110_46* | 1631 | Glutamine synthetase | Glutamine synthetase, catalytic domain | 1 |
| 3 | *NODE_115_3* | 7429 | Acetyl-CoA carboxylase | Acetyl-coenzyme A carboxyltransferase, N-terminal; ClpP/crotonase-like domain; | 1 |
| 4 | *NODE_130_53* | 3678 | 6-phosphofructokinase subunit alpha | Phosphofructokinase domain; | 3 |
| 5 | *NODE_1323_4* | 1861 | Hypothetical protein | / | 2 |
| 6 | *NODE_1331_7* | 2378 | DNA polymerase eta subunit | / | 1 |
| 7 | *NODE_15_2* | 1637 | Glucoamylase | Translation protein SH3-like domain | 1 |
| 8 | *NODE_1511_11* | 1927 | NADH kinase pos5 | NAD kinase/diacylglycerol kinase-like domain; | 1 |
| 9 | *NODE_1511_79* | 1576 | Methionine aminopeptidase | Winged helix-turn-helix DNA-binding domain; | 1 |
| 10 | *NODE_17_1* | 3657 | Transcription initiation factor TFIID subunit 1 | / | 2 |
| 11 | *NODE_17_25* | 2293 | Beta-hexosaminidase | Chitobiase/beta-hexosaminidase domain 2-like; | 1 |
| 12 | *NODE_171_30* | 5681 | Alpha-mannosidase | Galactose mutarotase-like domain; | 1 |
| 13 | *NODE_176_9* | 1243 | Protein transport protein SEC13 | WD40-repeat-containing domain; | 2 |
| 14 | *NODE_181_174* | 2299 | tRNA (guanine9-N1)-methyltransferase | WD40/YVTN repeat-like-containing domain; | 1 |
| 15 | *NODE_19_9* | 1020 | Hypothetical protein | / | 1 |
| 16 | *NODE_198_20* | 1493 | SH3 domain-containing protein | PH domain-like; NECAP, PHear domain; | 1 |
| 17 | *NODE_2_38* | 1948 | Glutamate decarboxylase | Phosphate-dependent transferase, major region, subdomain 1; | 1 |
| 18 | *NODE_201_16* | 3292 | Trihydroxynaphthalene reductase | / | 1 |
| 19 | *NODE_228_20* | 1647 | Aldose 1-epimerase | / | 1 |
| 20 | *NODE_2303_57* | 4506 | Flocculation protein FLO11 | / | 1 |
| 21 | *NODE_2303_72* | 1299 | Poly(A) RNA polymerase protein cid1 | Polymerase, nucleotidyl transferase domain; | 1 |
| 22 | *NODE_2303_85* | 3930 | Golgi apyrase | / | 1 |
| 23 | *NODE_249_23* | 2656 | Peptide methionine sulfoxide reductase | Peptide methionine sulphoxide reductase MsrA; | 2 |
| 24 | *NODE_254_7* | 3281 | Serine/threonine-protein kinase srk1 | Protein kinase domain; | 1 |
| 25 | *NODE_274_12* | 2296 | Glutathione S-transferase 1 | / | 2 |
| 26 | *NODE_281_31* | 4241 | Ribosome biogenesis protein NSA2 | / | 1 |
| 27 | *NODE_29_18* | 1997 | Translocation protein SEC66 | / | 1 |
| 28 | *NODE_30_68* | 1673 | 37S ribosomal protein S24 | Ribosomal protein S24/S35, mitochondrial, conserved domain; | 1 |
| 29 | *NODE_32_30* | 1445 | Hypothetical protein | HotDog domain; | 1 |
| 30 | *NODE_320_3* | 2517 | Putative C2H2 finger domain-containing protein | C2H2 finger domain | 7 |
| 31 | *NODE_320_34* | 1106 | Methionyl-tRNA formyltransferase | Formyl transferase, N-terminal; | 3 |
| 32 | *NODE_374_8* | 2772 | Protein transport protein SEC23 | Ribosomal protein L11, C-terminal; Sec23/Sec24, helical domain; | 2 |
| 33 | *NODE_378_72* | 2630 | GTP-binding protein ypt2 | / | 1 |
| 34 | *NODE_38_86* | 1284 | Aflatoxin B1 aldehyde reductase | NADP-dependent oxidoreductase domain; | 3 |
| 35 | *NODE_388_20* | 2567 | Uncharacterized RING finger protein P8B7.23 | / | 1 |
| 36 | *NODE_403_52* | 2185 | Zinc finger protein ADR1 | Zinc finger, C2H2-like; Transcription factor domain; | 1 |
| 37 | *NODE_405_44* | 1254 | Ubiquitin-conjugating enzyme E2 | / | 1 |
| 38 | *NODE_408_35* | 4224 | Nucleoporin nsp1 | / | 3 |
| 39 | *NODE_414_6* | 2385 | H/ACA Ribonucleoprotein complex subunit 1 | Translation protein, beta-barrel domain; | 1 |
| 40 | *NODE_439_51* | 338 | peptidase S8 and S53 | Peptidase S8/S53 domain; | 1 |
| 41 | *NODE_441_27* | 3135 | Aconitate hydratase, mitochondrial | Aconitase/3-isopropylmalate dehydratase large subunit, alpha/beta/alpha domain; | 1 |
| 42 | *NODE_486_20* | 1561 | Glucoamylase | PH domain-like; Anillin homology domain; | 1 |
| 43 | *NODE_492_8* | 1321 | Hypothetical protein | / | 2 |
| 44 | *NODE_505_4* | 1197 | Mitochondrial distribution and morphology protein 34 | / | 8 |
| 45 | *NODE_507_12* | 3684 | Kinesin heavy chain | Kinesin motor domain; | 1 |
| 46 | *NODE_514_22* | 2044 | C2H2 type zinc finger domain-containing protein | / | 1 |
| 47 | *NODE_52_12* | 1268 | COP9 signalosome complex subunit 5 | JAB1/MPN/MOV34 metalloenzyme domain; | 1 |
| 48 | *NODE_522_14* | 2566 | CCR4-NOT transcriptional complex subunit CAF120 | / | 4 |
| 49 | *NODE_525_16* | 2763 | DNA replication regulator SLD3 | / | 1 |
| 50 | *NODE_525_9* | 471 | Asparagine synthetase domain-containing protein | Glutamine amidotransferase type 2 domain; | 1 |
| 51 | *NODE_558_28* | 1753 | Protein transport protein SEC31 | / | 1 |
| 52 | *NODE_606_27* | 3481 | Phospho-2-dehydro-3-deoxyheptonate aldolase | Galactose mutarotase-like domain; | 7 |
| 53 | *NODE_647_10* | 3155 | Methionyl aminopeptidase | Phosphoribulokinase/uridine kinase; | 3 |
| 54 | *NODE_689_14* | 1344 | Glucose-6-phosphate 1-dehydrogenase | Glucose-6-phosphate dehydrogenase, C-terminal; | 4 |
| 55 | *NODE_69_11* | 4938 | Glucosamine-6-phosphate deaminase | p53-like transcription factor, DNA-binding; | 1 |
| 56 | *NODE_98_38* | 3556 | Elongation factor 2 | P-loop containing nucleoside triphosphate hydrolase; | 1 |
| 57 | *NODE_98_59* | 2013 | Splicing factor 1 | / | 1 |
| 58 | *NODE_98_63* | 3822 | DENN domain-containing protein | uDENN domain; DENN domain; | 2 |
| 59 | *NODE_99_2* | 2446 | DNA repair protein rad14 | / | 1 |
| 60 | *NODE_990_5* | 298 | Hypothetical protein | / | 1 |
